# Miocene spider* Maevia eureka* nov. sp. (Araneae: Salticidae)

**DOI:** 10.7717/peerj.3614

**Published:** 2017-07-25

**Authors:** Francisco Riquelme, Miguel Menéndez-Acuña

**Affiliations:** 1 Laboratorio de Sistemática Molecular, Escuela de Estudios Superiores del Jicarero, Universidad Autónoma del Estado de Morelos, Jojutla, Morelos, Mexico; 2 Maestría en Biología Integrativa de la Biodiversidad y la Conservación, Centro de Investigación en Biodiversidad y Conservación, Universidad Autónoma del Estado de Morelos, Cuernavaca, Morelos, Mexico

**Keywords:** Chiapas amber, Miocene, Totolapa, Salticidae, Maevia

## Abstract

A new fossil species of salticid spider (Araneae: Salticidae) is described based on an amber-embedded specimen. The specimen was collected from lignite-sandstone early-mid Miocene sediments near the town of Totolapa in Chiapas, southwestern Mexico. The diagnosis and description is supported by key characters that best match the genus *Maevia* Koch, 1846. Thus, this new fossil species has been named *Maevia eureka* nov. sp. This fossil shows closer affinities in epygine traits with extant specimens grouped around the species *Maevia poultoni* Peckham & Peckham, 1901. This represents the first known fossil species within *Maevia* and the southernmost record of the genus in North America that shows Nearctic relationships.

## Introduction

Fossils of jumping spiders (Araneae: Salticidae) are listed elsewhere (Dunlop, Penney & Jekel, 2017). The oldest known putative salticid form is considered to be from New Jersey amber, late Cretaceous (Grimaldi, Engel & Nascimbene, 2002). But the taxonomic identity of the presumed salticids from the Cretaceous period have been considered to be misidentified (Penney, 2007), including those from the Cretaceous amber of France and Jordan (Penney, 2010).

The indisputable fossil record of salticids is that of the Cenozoic of Europe (Paleogene) and the Americas (Neogene) (Dunlop, Penney & Jekel, 2017). The significant diversity of fossil salticids found in the Early to Middle Cenozoic is correlated positively to the fossilization in amber (Petrunkevitch, 1958; Prószynski & Zabka, 1980; Wolff, 1990; Wunderlich, 2004; Penney, 2008; Wunderlich, 2011). Therefore, all of the fossil salticids known from amber inclusions in the Paleogene are mostly from the Baltic amber-type localities in Europe, which include 12 genera with 41 described species and two undetermined taxa (Dunlop, Penney & Jekel, 2017). In the Americas, there are eight genera with 11 described species and four undetermined taxa in younger Neogene Dominican amber deposits (Penney, 2008; Dunlop, Penney & Jekel, 2017); whereas only three fossil specimens (*Lyssomanes* sp., incertae sedis and *Maevia* sp.) have been previously reported in Mexican amber (Petrunkevitch, 1971; García-Villafuerte & Penney, 2003; Riquelme & Hill, 2013).

The fossil studied herein was first reported as *Maevia* sp. in a previous contribution (Riquelme & Hill, 2013). The purpose of the present study is to give a full diagnosis and description of this salticid specimen, which best matches the extant genus *Maevia* Koch, 1846 recently assigned to the Subtribe Marpissina Simon, 1901 (Maddison et al., 2014; Maddison, 2015). Due to this, this fossil specimen is referred to here as *Maevia eureka* nov. sp.

The family Salticidae is a highly diversified and widespread group. A new comprehensive classification of Salticidae based on molecular and morphological data has been recently proposed (Maddison, 2015). Accordingly, the Subtribe Marpissina Simon, 1901 was placed within the Tribe Dendryphantini Menge 1879, *stat. nov*. Marpissina comprises 110 species in 9 genera, including the genus *Maevia* Koch 1846, and shows an almost exclusively New World distribution (Maddison, 2015).

*Maevia* was first placed in the subfamily Marpissinae with four other genera: *Paramaevia* Koch 1846, *Menemerus* Simon 1868, *Marpissa* C.L. Koch 1846, and *Metacyrba* Pickard-Cambridge 1901 (Barnes, 1958). Later, *Paramaevia* was moved back to *Maevia* (Edwards, 1977; Richman, Edwards & Cutler, 2005; Edwards & Hill, 2008). Also, *Menemerus* was recently included in the tribe Chrysillini (Maddison, 2015), and the genus *Metacyrba* was split in two genera: *Metacyrba* and *Platycryptus* (Hill, 1979) (Hill, 1979; Edwards, 2005). At present, using complementary molecular data, *Maevia* is considered to be a member of the subtribe Marpissina, which includes the genera *Marpissa*, *Mendoza* Peckham and Peckham, 1894, *Balmaceda* Peckham and Peckham, 1894, *Empanda* Simon, 1903, *Fuentes* Peckham and Peckham, 1894, *Metacyrba, Platycryptus* and *Psecas* C.L. Koch, 1850 (Maddison & Hedin, 2003; Maddison, 2015).

Thus, *Maevia* is a small taxon that mostly has a North American distribution, with several records in the United States of America. In addition, the species *Maevia inclemens* Walckenaer, 1837 is also located in Canada and *Maevia poultoni* Peckham and Peckham, 1901 has been recorded in northern Mexico (Barnes, 1955; Richman, Cutler & Hill, 2011). There are three presumed species reported in Peru and two in Sumatra (Taczanowski, 1878; Van Hasselt, 1882), but the Peruvian specimens listed by Taczanowski (1878) and the two species from Sumatra reported by Van Hasselt (1882) cannot be located, their taxonomic statuses are ambiguous, and their descriptions are limited. These taxa are mainly supported by color-based features with no images or data regarding the palp or epigynum. Initially, Taczanowski (1878) stated that specimens of *Maevia gracilipes, M. susiformis and M. trilineata* resemble those of *Maevia fenestrata* and *Maevia stolzmanni*; afterwards both *M. fenestrata* and *M. stolzmanni* were placed in the genus *Cotinusa* Simon, 1900. Therefore, it is reasonable to consider that the missing specimens from Peru most closely resembled *Cotinusa* rather than *Maevia* (Taczanowski, 1878). The presumed species from both Sumatra and Peru underscore the need for revision that clarifies their taxonomical identity. This conclusion applies to the other extant Australasian species, which have been tentatively included in *Maevia* but are currently *nomina dubia* (World Spider Catalog, 2017).

Living species of *Maevia* accentuate the differences between them with modified genitalia structure in both males and females, even more than other genera that also include diagnostic characters associated with other body structures. Accordingly, Barnes (1955) has initially proposed two separate subgenera on the basis of genitalia. Diagnostic characters for females are the shape and size of the epigynal copulatory opening and for males, the embolus morphology (Barnes, 1955). The so-called *‘M. inclemen* s’ group (reported for the USA and Canada only), first suggested as the subgenus *Maevia* by Barnes (1955), has an epigyne with a single, small, rounded copulatory opening; whereas the ‘*M. poultoni*’ group (also reported in Mexico), proposed as the subgenus *Paramaevia*, shows a epigyne with a large single median copulatory opening (Barnes, 1955). The new fossil species *M. eureka,* represented by an adult female, has an epigynum morphology closest to forms of ‘*M. poultoni*’ group, formerly known as *Paramaevia.*

### Locality and paleoenvironment

**Figure 1:**
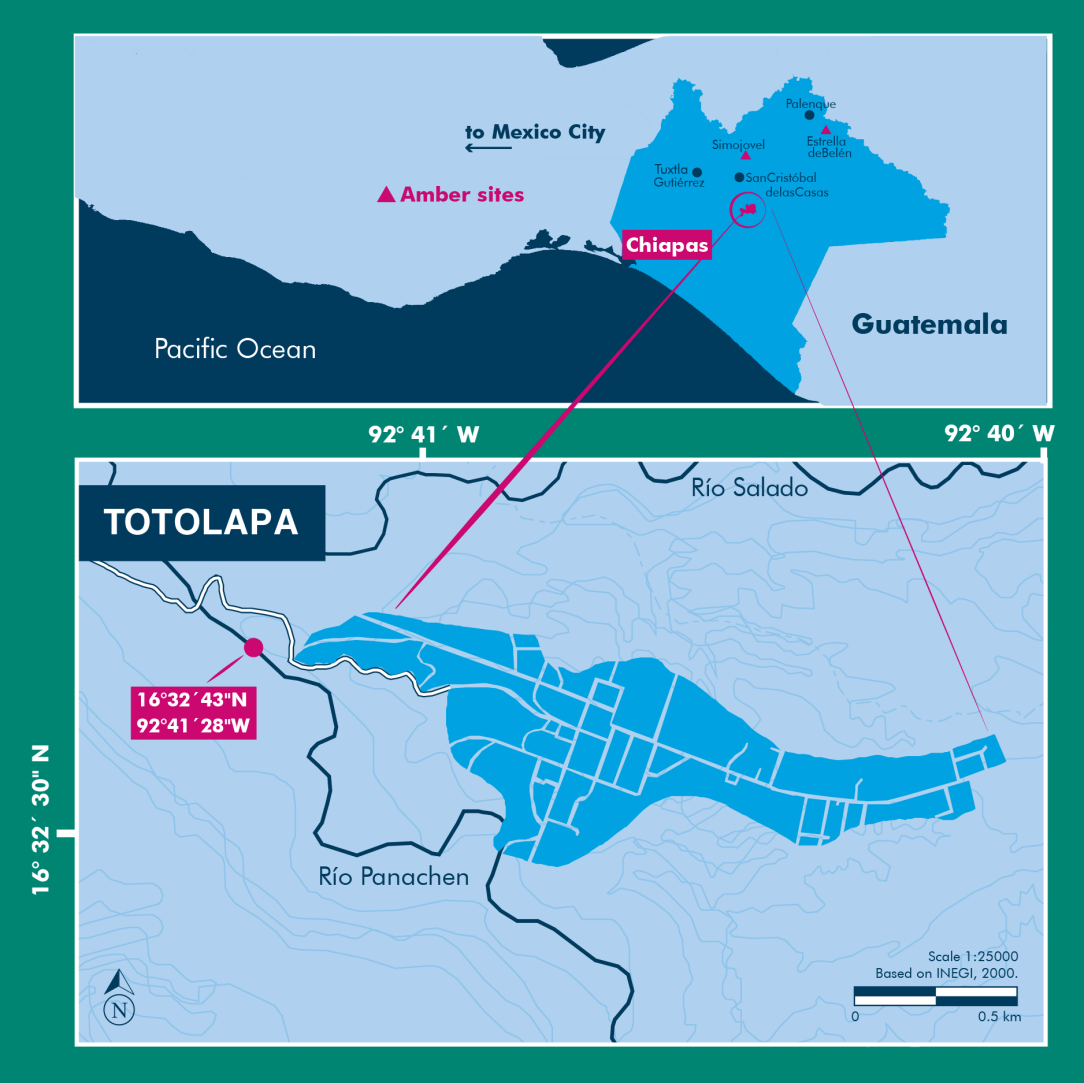
Location of the Río Panachen site near Totolapa, Chiapas, Mexico.

The amber specimen with the spider inclusion is a consolidated resin, with a golden yellow color, translucent glossiness, and secondary recrystallization with reddish marks, microfractures, and vesicles produced by volatile compounds released during resin hardening. Soil, microbial mats, insect parts, and plant debris are also present within the specimen. This amber specimen was recovered in sediments from the Río Panachen near the town of Totolapa (Fig. 1), one of the earliest amber sites in Chiapas, known since the pre-Columbian times (Lowe, 2004; Riquelme & Hill, 2013; Riquelme et al., 2014a). Totolapa sites have been inactive for a long period of time, but in recent years there has been a flurry of activity connected with amber extraction and trade (Bryant, 1983; Lowe, 2004). The stratigraphic position and lithology of Totolapa deposits are strongly associated with amber outcrops from Simojovel (Durán-Ruiz et al., 2013; Riquelme et al., 2014a). Simojovel sediments have been assigned to the Mazantic and Balumtum strata from early to mid-Miocene, c. 23-15 Ma (Perrilliat, Vega & Coutiño, 2010; Riquelme et al., 2014a). Recent paleontological fieldwork in Totolapa has preliminarily studied the rock section that contains the amber (Durán-Ruiz et al., 2013; Riquelme & Hill, 2013; Riquelme et al., 2014a; Breton, Serrano-Sánchez & Vega, 2014).

The source of Totolapa amber is the *Hymenaea* legume tree, similar to Simojovel amber (Lambert et al., 1989; Langenheim, 2003; Riquelme et al., 2014b). Amber-bearing beds from Totolapa, Simojovel, Huitupán and Estrella de Belén in the Chiapas Highlands are type localities of a well-known Konservat-Lagerstätte (Durán-Ruiz et al., 2013; Riquelme & Hill, 2013; Riquelme et al., 2014a; Riquelme et al., 2014b; Riquelme et al., 2014c). The sedimentary record and associated paleobiota suggests that the resin-producing trees of *Hymenaea* have been growth in a lowland-fluvial environment close to coastal plain (Graham, 1999; Langenheim, 2003; Perrilliat, Vega & Coutiño, 2010).

## Material and Methods

The amber inclusion studied here was collected from the lignite-sandstone beds exposed near Río Panachen in the town of Totolapa, 66 km south of Simojovel, State of Chiapas, Mexico. Preliminary, the collecting site was mistakenly identified in the first expedition as Río Salado (Riquelme & Hill, 2013). The holotype was designated BLMACH9 and deposited in the Museo del Ámbar de Chiapas (MACH), located in San Cristóbal de Las Casas, Chiapas, Mexico. The fossil amber collection at the MACH is formally certified by the Instituto Nacional de Antropología e Historia (INAH), a federal institute responsible for the archeological and paleontological heritage in Mexico. The export of fossils from Mexico without INAH registration certificate is illegal under federal laws. No specific permits were required for the specimen description.

Anatomical data were collected using high-resolution microscopy and multiple image-stacking for three-dimensional focus expansion (Riquelme et al., 2014a; Riquelme et al., 2014c). All photomicrographs were edited with Corel Paint X7^®^. Schematic drawings were hand traced by electronic pen using a stereomicroscope and photomicrographs and Corel Draw X7^®^ was used for graphic processing. Anatomical measurements are presented in millimeters and were collected using the open-source program tpsDig V. 2.17 (Rohlf, 2013).

The following anatomical abbreviations are used in this study: AME, Anterior median eyes; ALE, Anterior lateral eyes; PME, Posterior median eyes; PLE, Posterior lateral eyes; OQ, Ocular quadrangle; AER, Anterior eye row. For Macrosetation: tb, Tibia; mt, Metatarsus; v, ventral; pl, prolateral; rl, retrolateral.

The electronic version of this article in Portable Document Format (PDF) will represent a published work according to the International Commission on Zoological Nomenclature (ICZN), and hence the new names contained in the electronic version are effectively published under that Code from the electronic edition alone. This published work and the nomenclatural acts it contains have been registered in ZooBank, the online registration system for the ICZN. The ZooBank LSIDs (Life Science Identifiers) can be resolved and the associated information viewed through any standard web browser by appending the LSID to the prefix http://zoobank.org/. The LSID for this publication is: urn:lsid:zoobank.org:pub:52EFD563-69F6-41EE-B02C-D5C0C26562EE. The online version of this work is archived and available from the following digital repositories: PeerJ, PubMed Central and CLOCKSS.

## Results and Discussion

### Systematic paleontology

**Table:** 

Class **Arachnida** Cuvier, 1812
Order **Araneae** Clerck, 1757
Family **Salticidae** Blackwall, 1841
Genus ***Maevia*** Koch, 1846

***Maevia eureka*** nov. sp. Riquelme et Menéndez
ZooBank LSID: urn:lsid:zoobank.org:act:549ECDEF-8D66-43B3-BAA9-9603257997D5

**Figure 2:**
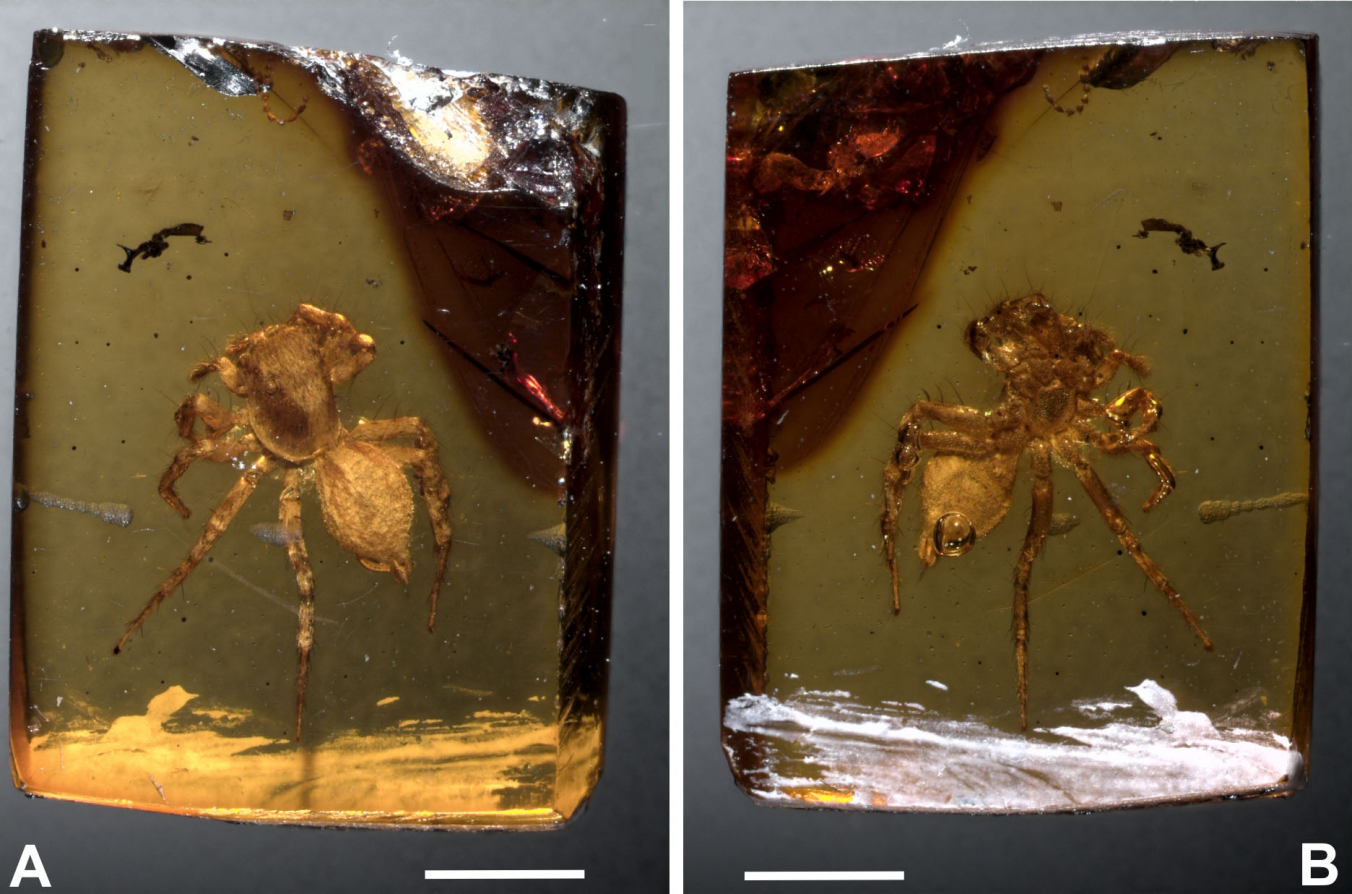
*Maevia eureka* nov. sp. Amber inclusion as seen in raw condition using regular light. (A) dorsal view. (B) ventral. Scale bar 2 mm.

**Figure 3:**
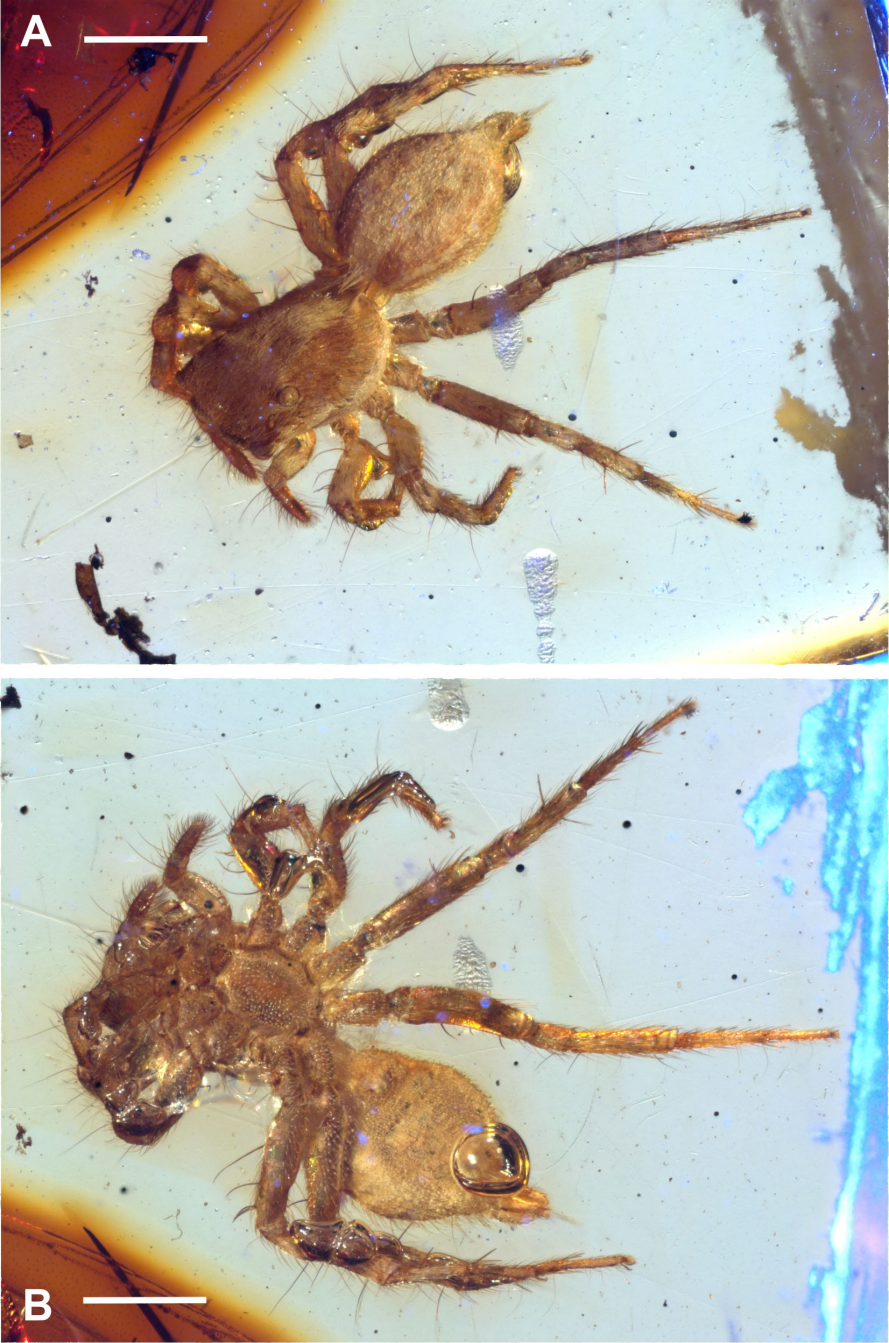
*Maevia eureka* nov. sp. Complete fossil specimen in closer view. (A) dorsal. (B) ventral. Scale bar 1 mm.

**Figure 4:**
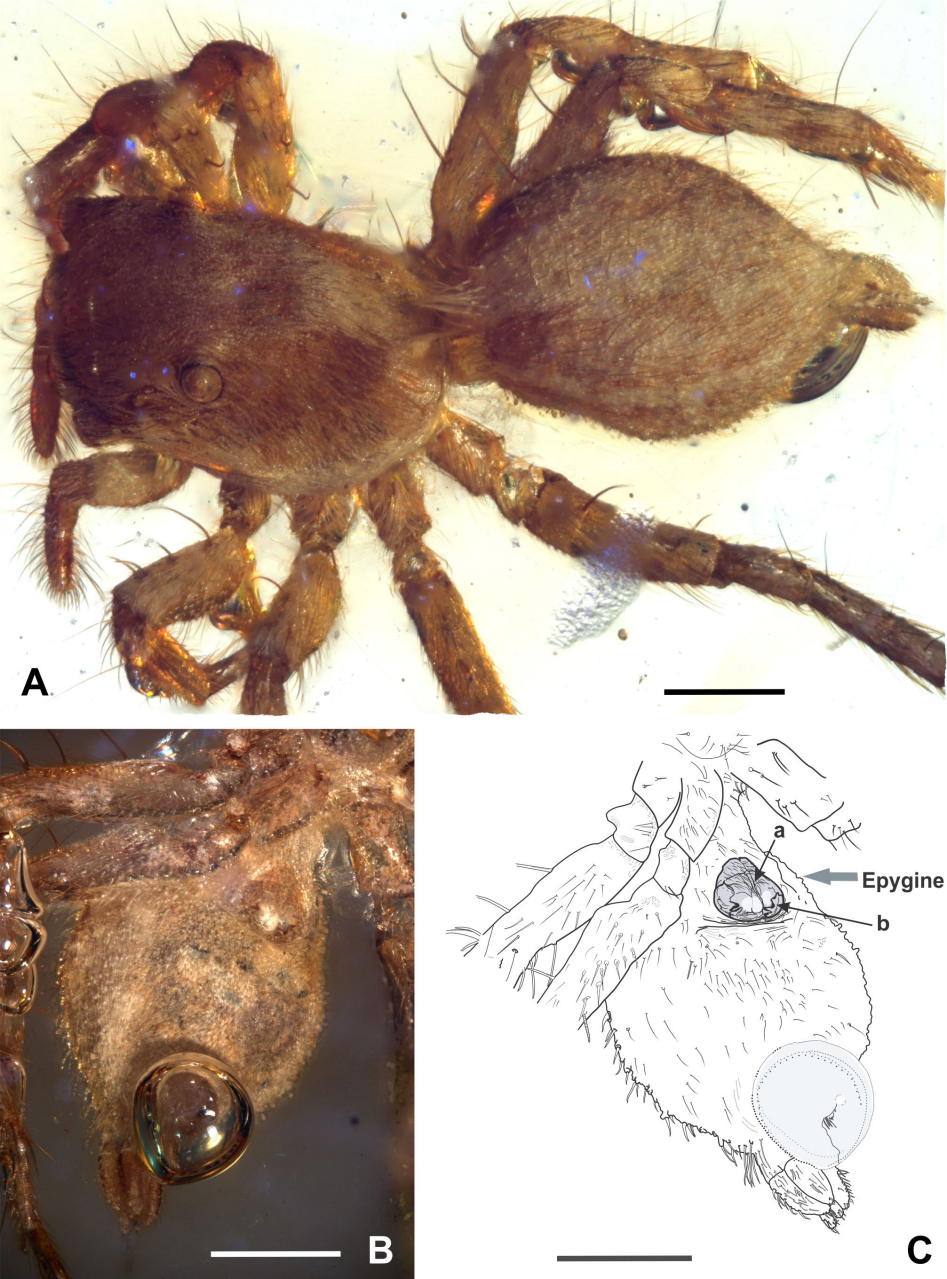
*Maevia eureka* nov. sp. (A) cephalothorax and abdomen in dorsal view. (B) abdomen in ventral view showing the epigyne. (C) schematic representation of the epigyne, a, copulatory opening; b, an M-shaped structure. Scale bar: 0.5 mm.

Diagnosis: *M. eureka* nov. sp. differs from all other congeners by having a combination of epigyne traits, such as a drop-shaped epigyne, almost as wide as long, with a single, large copulatory opening, located from the medial to the lower part of the epigyne, near the epigastric furrow (Figs. 2–4). *M. eureka* nov. sp can be distinguished from *M. inclemens*, *M. intermedia* and *M. expansa* by having a single, large copulatory opening, and differs from *M. poultoni*, *M. hobbsae* and *M. michelsoni* by the position of the copulatory opening in a lower part of the epigyne.

Derivation of name: named for the eureka moment of discovering the little amber piece during paleontological fieldwork, which was recovered in a hole dug by hand that was intended to be used as a toilet in the field.

Type material: Holotype BLMACH9, amber inclusion, entire adult female and only specimen known (Figs. 2–3). Currently deposited in the Museo del Ámbar de Chiapas (MACH), located in San Cristóbal de Las Casas, Chiapas, Mexico.

Locality and Horizon: Río Panachen, latitude 16°32′43″N, longitude 92°41′28″W, located near the town of Totolapa, Chiapas, Mexico. The amber-bearing beds in Totolapa are associated with the Mazantic shale and Balumtum sandstone strata from the Simojovel sites, which are dated initially as early-mid Miocene in age (Perrilliat, Vega & Coutiño, 2010; Durán-Ruiz et al., 2013; Riquelme et al., 2014a).

Taphonomic features: Well-articulated fossil specimen, buried in amber, with remarkable fossil preservation of hard tissues and scales. Dorsally, the true color morphology is seen clearly. Ventrally, there is a single bubble projecting from the anus, most likely from gas released as the specimen died. The delicate preservation of such a feature is indicative of rapid resin hardening (Riquelme et al., 2014b). A few anatomical elements cannot be seen as they are minute and covered by bubbles or body posture (i.e., some spines on tibia from leg I). Although the fossil body is structurally intact, there is also a significant physical thinning and clearing of the cuticle observable. This is probably caused by long-term chemical reactions within the resin that contains organic acids (Figs. 2A–2B).

Color of specimen preserved in amber*:* body uniformly pale brown, integument of prosoma brown with a longitudinal band of white scales running from the OQ to the pedicel in dorsal view. Other longitudinal white bands occur in lower lateral side of carapace, OQ darker, elevated from the prosoma. The abdomen is predominantly pale brown, with a wide white dorsal band and two narrow white lateral bands that extend from the pedicel to spinnerets. Legs are pale brown in general (Figs. 3A–3B).

Description: Small-sized salticid, adult female, body 3.6 mm of total length without chelicerae, fossil inclusion (Figs. 3A–3B).

Carapace: 1.5 long, 1 wide. OQ 0.5 long; AER 0.9, PER 0.6. Eye diameter 0.2 AME, 0.1 ALE, 0.05 PME, 0.1 PLE, distance between ALE-PME 0.1, PLE-PME 0.1. Small depression behind OQ. Brown setae covering most of the body, predominantly the legs, the tibia and tarsus of the palp. Chelicerae with two small fangs only slightly visible in ventral view. Endites quadrangular, convergent apically, labium triangular, as wide as long, 2/3 the length of endites. Sternum longer than wide, anteriorly almost as wide as labium (Figs. 3A and 4A).

Abdomen: ovoid, 1.8 long, 1.1 wide, pale brown, dorsally a wide longitudinal white band and laterally two narrow longitudinal white bands running from pedicel to spinnerets, covered by abundant brown setae and a distinctive tuft of white setae running from the abdomen to the carapace, covering the pedicel in dorsal view. Two anterior lateral and two posterior lateral spinnerets, cylindrical, covered by brown setae. Venter pale brown, tinged with abundant white scales and some black spots (Fig. 3B).

Legs: pale brown in general, femur I and II with dorsal white scales, all femora with 2–4 brown spines dorsally. Leg formula IV: III : I : II, always ending in two claws, claws tufts present. Length of coxa I 0.2, II 0.3, III 0.4, IV 0.4; trochanter I 0.2, II 0.1, III 0.1, IV 0.2; femur I 0.5, II 0.4, III 0.7, IV 0.8; patella + tibia I 0.5, II 0.5, III 0.9, IV 1; metatarsus + tarsus I 0.6, II 0.4, III 0.8, IV 1. Leg spines variable, with a set of spines at the junction of the metatarsus and tarsus is easily seen in both leg III and IV. The following macrosetation pattern is observable: Leg I, tb 2v; Leg II, tb 2rl-2v; mt 4v. Leg III, tb 1pl-1rl; mt 2v-1rl-1pl. Leg IV, tb 1pl-1rl; mt 2v-1rl-1pl (Figs. 3A and 3B).

Epigyne: well-sclerotized, drop-shaped, adjacent to the epigastric furrow, almost as wide as long, 0.3 long, 0.2 wide, brown color with a white spot located in the middle-bottom; with a single, large epigynal copulatory opening from the medial to the lower part of the epigyne, near the epigastric furrow, with an M-shaped structure visible at each side (Figs. 4B–4C).

## Remarks

This fossil was placed within *Maevia* on the basis of diagnostic characters shared with the living species of this genus. Thus, *M. eureka* nov. sp. shows close affinities with *Maevia inclemens* Walckenaer, 1837, *Maevia poultoni* Peckham and Peckham, 1901, *Maevia expensa* Barnes, 1955, *Maevia hobbsae* Barnes, 1955, *Maevia intermedia* Barnes, 1955, and *Maevia michelsoni* Barnes, 1955; such as the lateral margins of the carapace rounded with the widest point behind de PLE, the PME halfway between PLE and ALE, the carapace height at least 60–70% if its greatest width, the ocular quadrangle occupying nearly 40 percent of total carapace length, the first pair of legs not markedly heavier than the others, and the abdomen almost as wide as it is long (Barnes, 1955; Barnes, 1958; Hill, 1979; Logunov, 1999; Logunov & Cutler, 1999; Edwards, 2005). As well as the general form of the epigyne that best matches *M. poultoni* (Barnes, 1955), suggesting that living forms closest to *M. poultoni* might possibly have a shared ancestral homology with the fossil *M. eureka* nov. sp.

In addition, the general appearance of the body of *M. eureka* nov. sp. superficially resembles the unrelated females of the genus *Thiodina* (=*Colonus*), which is also observable in the extant female forms of *M. inclemens* as first stated by Edwards & Hill (2008), but it clearly differs by body size, tibia I, dark/clear longitudinal bands on the abdomen, as well as the shape of the epigyne and copulatory openings as described by Richman & Vetter (2004); females of *Thiodina* (=*Colonus*) has two copulatory openings while *M. eureka* nov. sp. have only one. Also, the epigyne in *M. eureka* nov. sp. somewhat resembles living forms of the genera *Freya, Frigga, Fuentes* and *Paramarpissa* but is easily distinguished by having a single copulatory opening, instead of the two copulatory openings as presented in both *Freya* and *Frigga* (Edwards, 2015); and it clearly differs from *Fuentes* and *Paramarpissa* by having the abdomen almost as wide as long, the first pair of legs not markedly heavy, and different spination on tibia I (Logunov & Cutler, 1999; Ruiz & Brescovit, 2006).

*M. poultoni* was considered the type species of the subgenus *Paramaevia* Barnes (1955) and Barnes (1958)*,* but further discussions placed *Paramaevia* as synonymous of *Maevia* (Edwards, 1977; Richman, Edwards & Cutler, 2005; Edwards & Hill, 2008). According to the literature, the genus *Maevia* was initially separated in two groups: the subgenera *Maevia* and *Paramaevia*, based on differences present in male palp (embolus) and female epigynum (copulatory opening) (Barnes, 1955; Barnes, 1958). The ‘*Maevia*’ group includes forms with a thin long embolus and tiny median openings in the epigynum closely related with *M. inclemens*. In contrast, the ‘*Paramaevia*’ group includes forms with a heavy embolus and epigynum with large median circular copulatory openings more closely related with *M. poultoni* (Barnes, 1955). The fossil *M. eureka* nov. sp. resembles females from the ‘Paramaevia’ group (i.e., those species closest to *M. poultoni*) such as *Maevia hobbsae* (Barnes, 1955) and *Maevia michelsoni* (Barnes, 1955). However, *M. eureka* nov. sp. is easily distinguished from all other congeners by a combination of epigyne traits, such as a drop-shaped epigyne, almost as wide as long, with a single, large copulatory opening from the medial to the lower part of the epigyne, near the epigastric furrow. *M. eureka* nov. sp. can be distinguished from *M. inclemens*, *M. intermedia* and *M. expansa* by the single, large copulatory opening and separates from *M. poultoni*, *M. hobbsae* and *M. michelsoni* by the position of the copulatory opening in a lower part of the *epigyne* (Figs. 2–4). In addition, an M-shaped structure is observable at each side of the epigyne copulatory opening in *M. eureka* nov. sp. which might be considered as the possible site for the male RTA to attach. This feature was revealed due to physical thinning and clearing of the cuticle by the long-term chemical reactions between the spider body and plant resin.

On the other hand, the four pairs of ventral spines on the tibia on leg I as seen in *M. inclemens* is not conclusively seen in the present fossil specimen. Barnes (1955) initially stated that the leg spines in *Maevia* could be variable, but conventionally, the tibia on leg I show four pairs of ventral spines. However, Barnes (1955) deliberately omitted this character in the formal diagnosis from both *M. expansa* and *M. intermedia*. There are two pairs of ventral spines in *M. eureka* nov. sp. that are clearly visible. Unfortunately, the other pairs of ventral spines associated with *M. inclemens* as seen in Barnes (1955) cannot be unambiguously confirmed in the amber-embedded specimen, because legs I and II are tightly folded and covered by bubbles.

## Conclusion

No descriptions of fossil species in *Maevia* are known to date. The evolutionary patterns of the genus *Maevia* are still unclear. The last review of *Maevia* identifies living species of North America (Barnes, 1955; Richman, Cutler & Hill, 2011), which includes all the six valid species present in the USA. The species *M. inclemens* is also found in Canada and *M. poultoni* is documented in Mexico, specifically, at the northern State of Tamaulipas (Richman, Cutler & Hill, 2011). Thus, the fossil *M. eureka* nov. sp. is the southernmost record of the genus *Maevia* in North America.

The fossil record of salticids in Chiapas amber comprises two undetermined specimens (Petrunkevitch, 1971; Dunlop, Penney & Jekel, 2017), and one juvenile specimen reported as putative member of the genus *Lyssomanes* (García-Villafuerte & Penney, 2003). Therefore, *M. eureka* nov. sp. is the first described species of Salticidae in Chiapas amber. The family Salticidae from Mexico currently comprises 65 genera and 264 valid species (World Spider Catalog, 2017). There are 14 genera and 25 living species recorded in the State of Chiapas, none belongs to *Maevia* (Richman, Cutler & Hill, 2011; Garcilazo-Cruz & Alvarez-Padilla, 2015).

The occurrence of *M. eureka* nov. sp. provides additional paleobiogeographical insights into living populations of North America. As mentioned above, the new fossil species *M. eureka* shows closer affinities in the epygine traits with the living specimens grouped around the *M. poultoni* or the so-called ‘*Paramaevia’* group. Therefore, the current forms closest to *M. poultoni* likely share an ancestral homology with *M. eureka* nov. sp. This fossil record from southern Mexico (Chiapas) also agrees with another record of living species from northern Mexico (Tamaulipas) belonging to the species *M. poultoni*. This probably indicates that *Maevia* was dispersed throughout the Neotropics in the Miocene. Most likely, the genus eventually became extinct in the south but remained in the north almost exclusively.

Fossil salticids from Europe (Baltic amber: Paleogene) and Middle America (Dominican and Mexican amber: Neogene) distributed in disconnected continents during early to mid-Cenozoic could suggest that a common origin of salticids prior to the separation of the continents (Mesozoic) is plausible. However, fossils confidently assignable to the family Salticidae have so far been found exclusively in the early to mid-Cenozoic (Penney, 2007; Penney, 2010; Selden & Penney, 2010; Dunlop, Penney & Jekel, 2017). In consecutive molecular phylogenetic analyses performed by Maddison & Hedin (2003) and Maddison et al. (2014), including molecular clock analyses calibrated by Cenozoic amber fossil data (Bodner & Maddison, 2012), it has been suggested that major clades of Salticidae are mostly restricted to particular continental regions and their radiations probably postdate the Mesozoic breakup, although some confidence intervals on divergence estimates go into the Cretaceous (see Table 1 in Bodner & Maddison, 2012). Accordingly, the fossil record is consistent with a Cenozoic origin of Salticidae as suggested by molecular data, but is also consistent with a pre-Cenozoic origin that agrees with the general geological history of the continents. In the Americas, a more intense radiation of the New World clades probably took place from the Neogene (Cenozoic) as suggested by the fossil record in the Middle America and the diversity at the genus and species level found today in the Tropics and North America as preliminary noted by Hill & Richman (2009) and Hill & Edwards (2013). Thus, the discovery of the present fossil *M. eureka* nov. sp. is consistent with a subsequent radiation in the Neogene with Nearctic relationships.

##  Supplemental Information

10.7717/peerj.3614/supp-1 Supplemental Information 1 Holotype repository The holotype was designated BLMACH9 and deposited in the Museo del Ámbar de Chiapas (MACH), located in San Cristóbal de Las Casas, Chiapas, Mexico.

## Additional Information and Declarations

### Data Availability

The following information was supplied regarding data availability: The specimen is currently deposited in the Museo del Ámbar de Chiapas (MACH).

### New Species Registration

The following information was supplied regarding the registration of a newly described species: Publication LSID: urn:lsid:zoobank.org:pub:52EFD563-69F6-41EE-B02C-D5C0C26562EE Species name: urn:lsid:zoobank.org:act:549ECDEF-8D66-43B3-BAA9-9603257997D5.
