# Supplementary material for: Miocene spider Maevia eureka nov. sp. (Araneae: Salticidae)
Source: PeerJ. 2017 Jul 25;5:e3614. doi: 10.7717/peerj.3614 (PMC5530986; doi:10.7717/peerj.3614)
Supplement: Supplemental Information 1 — The holotype was designated BLMACH9 and deposited in the Museo del Ámbar de Chiapas (MACH), located in San Cristóbal de Las Casas, Chiapas, Mexico. [file peerj-05-3614-s001.docx]

The holotype was designated BLMACH9 and deposited in the Museo del Ámbar de Chiapas (MACH), located in San Cristóbal de Las Casas, Chiapas, Mexico. The fossil amber collection at the MACH is formally certified by the Instituto Nacional de Antropología e Historia (INAH), a federal institute responsible for the archeological and paleontological heritage in Mexico**.**
